# Quorum Sensing System-Regulated Proteins Affect the Spoilage Potential of Co-cultured *Acinetobacter johnsonii* and *Pseudomonas fluorescens* From Spoiled Bigeye Tuna (*Thunnus obesus*) as Determined by Proteomic Analysis

**DOI:** 10.3389/fmicb.2020.00940

**Published:** 2020-05-14

**Authors:** Xin-Yun Wang, Jing Xie

**Affiliations:** ^1^Shanghai Engineering Research Center of Aquatic Product Processing & Preservation, Shanghai Ocean University, Shanghai, China; ^2^National Experimental Teaching Demonstration Center for Food Science and Engineering, Shanghai Ocean University, Shanghai, China; ^3^College of Food Science and Technology, Shanghai Ocean University, Shanghai, China

**Keywords:** quorum sensing, autoinducers (AHLs), AI-2, proteome, *Acinetobacter johnsonii*, *Pseudomonas fluorescens*, spoilage

## Abstract

Food spoilage by certain species of bacteria is reported to be regulated by quorum sensing (QS). *Acinetobacter johnsonii* and *Pseudomonas fluorescens*, the major specific spoilage organisms, are found to be limited in their QS and co-culture interactions. The aim of this study was to determine how QS-regulated proteins affect the spoilage potential of co-cultured *A. johnsonii* and *P. fluorescens* obtained from spoiled bigeye tuna (*Thunnus obesus*) using a proteomics approach. The *A. johnsonii, P. fluorescens*, and their co-culture tested the *N*-acyl-homoserine lactone (AHL) activities using reporter *Chromobacterium violaceum* CV026 and LC-MS/MS in qualitative and quantitative approaches, respectively. These latter showed that, of the 470 proteins and 444 proteins in *A. johnsonii* (A) and *P. fluorescens* (P), respectively, 80 were significantly up-regulated and 97 were significantly down-regulated in A vs. AP, whereas 90 were up-regulated and 65 were down-regulated in P vs. AP. The differentially expressed proteins included the AI-2E family transporter OS, 50S ribosomal protein L3, thioredoxin reductase OS, cysteine synthase CysM OS, DNA-binding response regulator, and amino acid ABC transporter ATPase OS. The cellular process (GO:0009987), metabolic process (GO:0008152), and single-organism process (GO:0044699) were classified into the gene ontology (GO) term. In addition, energy production and conversion, amino acid transport and metabolism, translation, ribosomal structure and biogenesis, post-translational modification, protein turnover, and chaperones were distributed into the clusters of orthologous groups of proteins (COG) terms. The KEGG pathways revealed that 84 and 77 differentially expressed proteins were divided into 20 KEGG pathways in A vs. AP and P vs. AP, respectively, and amino acid metabolism, carbohydrate metabolism, energy metabolism, and translation were significantly enriched. Proteins that correlated with the spoilage-related metabolic pathways, including thioredoxin reductase OS, cysteine synthase OS, and pyridoxal phosphate-dependent enzyme family protein OS, were identified. AI-2E family transporter OS and LuxR family transcriptional regulator OS were identified that related to the QS system. These findings provide a differential proteomic profile of co-culture in *A. johnsonii* and *P. fluorescens*, and have potential applications in QS and the regulation of spoilage potential.

## Introduction

Bigeye tuna (*Thunnus obesus*) is a highly sought after fish species used to prepare sashimi in many countries around the world (Wang and Xie, 2019). However, bigeye tuna is easily spoiled by specific spoilage organisms during refrigerated storage, which leads to a reduced shelf life (Sun et al., 2013; Silbande et al., 2016). Currently, more attention has been paid to convenient methods of refrigeration for storing bigeye tuna than to how spoilage bacteria in refrigerated tuna develop through their interactions with each other (Wang et al., 2017). The microbial spoilage of aquatic products is correlated mainly with Gram-negative bacteria, including *Acinetobacter* spp., *Shewanella* spp., *Pseudomonas* spp., *Aeromonas* spp., lactic acid bacteria, and the Enterobacteriaceae family, when stored under different storage conditions. The main species of bacteria leading to the spoilage of aquatic products during cold storage are *Acinetobacter johnsonii* and *Pseudomonas fluorescens*, which are commonly referred to as specific spoilage organisms (SSOs) (Jia et al., 2018; Pang and Yuk, 2019; Zhu et al., 2019). It is a significant SSOs due to its ability to produce volatile sulfides, amines, trimethylamine extracellular enzymes, trimethylamines, organic acids and some spoilage metabolites.

Quorum sensing (QS) is the mechanism by which cell population-dependent signaling and interactions are recognized by bacteria in order to modulate their collective behaviors, including spoilage activity, enzyme secretion, bioluminescence, biofilm formation, virulence, and several signal molecules that mediate this mechanism have now been reported (Natrah et al., 2012; Zhu S. et al., 2015). There are various QS molecules, such as CAI-1 (cholera autoinducer 1), DKPs (Diketopiperazines), HAQs (4-hydroxy-2-alkylquinolines), DSFs (Diffusible Signal Factors), AI-2 (Autoinducer-2) and indole, etc (Monnet and Gardan, 2015; Papenfort and Bassler, 2016). QS-mediated communication is based on the prevailing interspecies communication in both Gram-negative and Gram-positive bacteria occurring via autoinducer-2 (AI-2) and auto-inducing peptides (AIPs) (Stephens et al., 2019). *P fluorescens* is a Gram-negative bacteria that has been reported to use *N*-acyl-homoserine lactone (AHL) signals to monitor its local population through the exchange of extracellular signal molecules (Li et al., 2018). SSOs utilize QS communication of circuits to regulate a diverse array of physiological activities microbial, including eavesdropping, biofilm genesis, and bioluminescence. Recently, several studies have found that QS signal molecules were *N*-butyryl-DL-homoserine lactone (C4-HSL), *N*-hexanoyl-DL-homoserine lactone (C6-HSL), octanoyl-L-homoserine lactone (C8-HSL), decanoyl-homoserine lactone (C10-HSL), and *N*-dodecanoyl-L-homoserine lactone (C12-HSL), which significantly reduced the protease activities and spoilage potential of SSOs. Moreover, QS systems can govern bacterial behavior in food spoilage ecology (Whiteley et al., 2017). Bacterial growth, protease production, spoilage potential, electrochemical tests, and spoilage protein expression were significantly enriched through AHL signal regulation (Zhu et al., 2017). However, few studies have focused on how two species of SSOs regulate their protein function in relation to spoilage potential.

Recently, proteomic analysis has become a beneficial and quantifiable technique for providing relative measurements of proteins from different samples by nano-liquid chromatography-tandem mass spectrometry (nano-LC-MS/MS) without any isotope labeling. In a previous study on QS signaling, an absolute quantitative proteomic experiment was designed to investigate how Cyclo (L-phenylalanine-L-proline) effects protein expression in *Staphylococcus aureus.* Cyclo (L-phenylalanine-L-proline) has focused mainly on LuxR-mediated QS systems in bacteria, while the mechanism of extracellular QS signal molecule remained known (Ai et al., 2019). Wang et al. (2019) identified 1103 acetylated proteins and 2929 acetylation sites in *Shewanella baltica* from aquatic products to evaluate spoilage activity by LC-MS/MS analysis. A label-free quantitative proteomic approach has been applied to analyze the differential protein expression of *Enterococcus faecalis* SK460 to determine potential factors related to enterococcal biofilm formation. The results of this study showed that the related protein in the relevance of LuxS QS and pheromone in the biofilm development of *E. faecalis* was due to the Fsr being lacking in QS (Suryaletha et al., 2019). Based on proteomic analysis, a total of 338 vesicular proteins of *Pseudomonas aeruginosa* were identified with high confidence by LC-MS/MS analysis. This proteome profile provides a basis for future studies to illustrate the pathological functions of outer membrane vesicles from *P. aeruginosa* (Choi et al., 2011). However, there are very few studies demonstrating the fact that two SSOs have a possible role in QS for controlling expression of their target proteins and regulating bacterial behavior.

The aim of this study was to examine AHL production and AI-2 activity in *A. johnsonii*, *P. fluorescens*, and their co-culture, and to examine AHL-based QS systems regulating biofilm formation, protease activity, spoilage potential, and key proteins. Proteomic analysis of *A. johnsonii*, *P. fluorescens*, and their co-culture was used to elucidate the potential role of QS systems and their expression of proteins, interspecies communication, and metabolic pathways, which might be useful for developing effective methods for detecting spoilage capability.

## Materials and Methods

### Preparation of Bacterial Strain Cultures

Two kinds of SSOs (*A. johnsonii* and *P. fluorescens*) were originally isolated from spoiled bigeye tuna muscle (Zhejiang Fenghui Ocean Fishing Co., Ltd., China) by 16s rRNA gene sequences and VITEK^®^ 2 CompactA system (bioMérieux, France). Stock cultures containing 25% glycerine were stored at −80°C. Before use, *A. johnsonii* and *P. fluorescens* were pre-cultured individually over two successive periods of 18 h in a brain-heart infusion broth at 30°C and then cultured in tryptic soya broth until the maximal concentration (10^8^ CFU/mL) was attained. The bioreactors were inoculated with single strains overnight culture at a 1% (v/v) inoculation level. The co-culture consisted of a mixture of equal amounts (v/v) of *A. johnsonii* and *P. fluorescens*. All bacterial strains were cultured overnight at 30°C, then centrifuged at 10,000 *g* for 10 min and collected in a 15 mL centrifuge tube to remove the medium. The precipitate was finally collected and washed three times with pre-cooled 10 mL 1 × PBS solution. The precipitate obtained was transferred to a new 2 mL centrifuge tube and immediately stored at −80°C. *Chromobacterium violaceum* CV026 and *Vibrio harveyi* BB170 were kindly donated by Dr. Mi (Bohai University, Jinzhou, China).

### Preparation of Reagents

C4-HSL, C6-HSL, and C8-HSL with purities of over 96% were purchased from Sigma-Aldrich (St.Louis, MO, United States). Other reagents were analytical grade and were purchased from Sangon Biotech Co., Ltd (Shanghai, China) and Aladdin Industrial Corporation (Shanghai, China).

### Detection of AHLs and AI-2 Activity

#### Detection of AHLs by Cross-Feeding Plate Assay

Detection of AHLs was carried out by the cross-feeding plate assay reported by Chu et al. (2011). Test strains (*A. johnsonii*, *P. fluorescens* and co-culture) were cultured with shaking in TSB at 30°C for 24 h. CV026 was cultured with shaking in Luria-Bertani (LB) broth supplemented with kanamycin (20 μg/mL) as the reporter strain at 30°C for 24 h. All strains were grown to approximately 10^8^ CFU/mL (OD600≈0.8). The test strain and CV026 were struck in parallel on the LB nutrient agar plate. The plate was dried with sterile air and then incubated at 28°C for 24 h. The reporter strain was used as the negative control.

#### Detection of AI-2 Activity

*Vibrio harveyi* BB170 was inoculated into the Autoinducer Bioassay (AB) liquid medium (Bodor et al., 2008) and cultured overnight with consistent shaking in AB liquid medium adjusted to 10^8^ CFU/mL (OD600≈0.8). The fresh AB medium and the test bacterial solution were mixed and diluted at a ratio of 1:5000. A volume of 20 μL of culture supernatant and 180 μL of *V.harveyi* BB170 dilution were mixed at 100 r/min for 4h at 30°C in a 96-well black microtiter plate. Finally, a light unit at a wavelength of 460 nm was measured every 1d with a multiplate reader (Synergy 2, BioTek, Winooski, VT, United States). The cell-free culture of *V. harveyi* BB170 supernatant was defined as a positive control. Sterile AB medium served as a blank control. This test was repeated three times.

### Extraction and Detection of AHLs by HPLC-MS

To acquire the supernatant, the concentrated bacteria of the test bacterial cultures during the logarithmic phase of growth was centrifuged at 10,000 r/min for 10 min at 4°C. A volume of 50mL of the supernatant was extracted three times into an equal volume of acidified ethyl acetate (0.5% acetic acid). The AHLs were dissolved in an appropriate volume of methanol and stored at −20°C for further experiments.

The solution of AHLs was drained of solvent, and then 1mL of the extracted solution (methanol:water = 80:20) was added to an ultrasound steel ball grinding machine (6 min, 50 Hz) at −20°C for 30 min and centrifuged at 13000 *g* for 15 min at 4°C to obtain 200 μL of supernatant.

Quantitative experiments involving the AHLs were performed on a Q Exactive HF-X mass spectrometer (Thermo Fisher Scientific, Waltham, MA, United States) coupled to an Easy-nLC 1200 nano-flow UHPLC. The chromatographic parameters were as follows: column: BEH C18 (100 × 2.1 mm i.d., 1.7 μm; Waters Corporation, Milford, MA, United States), flow rate: 0.4 mL/min, sample injection volume: 5 μL, gradient mobile phase: water with 0.1% formic acid as solvent A and 0.1% formic acid and methanol as solvent B, column temperature: 40°C, and ion mode: positive ion mode (ESI+). The gradient mobile phase program applied was as follows, *t* = 0 min, 90% A; *t* = 4 min, 5% A; *t* = 9 min, 5% A; *t* = 7.1 min, 90% A; *t* = 10 min, 90% A. The conditions of MS/MS included the ion source temperature: 320°C, spray voltage: 3.5kV, sheath gas flow rate: 50 Arb and MS/MS collision energy: 70–1050 V.

### Protease Activity Assay

The protease activity of the culture supernatant was determined according to the method described by Zhu J. et al. (2015), with a slight modification. A volume of 20 μL of the culture supernatant was added to 2.5 μL of the protease substrate solution. Incubation buffer was added to adjust the final assay volume to 50 μL. The tube was sealed and incubated at 37°C for 0.5 h. Following incubation, 50 μL of precipitation agent were added, and the contents were mixed and incubated again at 37°C for 10 min. The tubes were then centrifuged at 12,000 *g* for 5min. All experiments were conducted in triplicate.

### Biofilm Formation Assay

Biofilm formation was investigated using an alteration of the method reported by Li et al. (2018). Biofilm formation was measured by crystal violet assay. Briefly, the above 96-well polystyrene microtiter plates inoculated *A. johnsonii*, *P. fluorescens* and co-culture. Following incubation, the biofilm in the plates in each well was carefully washed thrice with sterile phosphate buffered saline (PBS, pH.0) to remove unattached cells. After drying, the wells were added 0.2% (w/v) crystal violet to stain 15 min. The wells were then washed and dried as described above, then the dye attached to the biofilm was re-solubilized with 95% ethanol for 5 min. A volume of 200 μL of the sample solution was measured at 595 nm using a microplate reader.

### Proteome Analysis

#### Protein Extraction and Digestion

The bacterial cells were mixed with lysis solution (8 M urea, protease inhibitor cocktail), and incubated in ice-bath for 30 min, vortex-oscillated for 5 s every 5 min. The mixture was centrifuged at 12,000 *g*, 4°C for 30 min to obtain supernatant. Protein concentrations were measured using a BCA Assay Kit (Thermo Fisher Scientific, United States). Each sample tube contained 150 μg of protein. The sample solution was added to tris(2-carboxyethyl)phosphine (TCEP) to reach a final concentration of 10 mM and incubated at 37°C for 60 min. Then, an appropriate quantity of iodoacetamide (IAM) was added to achieve a final concentration of 40mM and incubated for 40min in the dark. Finally, 100 mM triethylammonium bicarbonate (TEAB) buffer was added to dilute the concentration of the solution. To each sample tube was then added trypsin solution in the ratio 1:50, and the tubes were thenincubated at 37°C overnight.

#### Mass Spectrometry Analysis and Protein Identification

A total of nine samples from three groups (A, P, AP groups) were analyzed on a Q Exactive HF-X mass spectrometer coupled to an Easy-nLC 1200 nano-flow UHPLC. Three biological replicates were used per sample. Each peptide sample (0.5 μgμL) was injected for nano-LC-MS/MS analysis. Each sample was loaded onto the C18 reversed phase column (75 μm × 25 cm, Thermo Fisher Scientific, United States) having two solvent systems (buffer A: 2% acetonitrile and 0.1% formic acid; buffer B: 80% acetonitrile and 0.1% formic acid) for 160 min at a flow rate of 300 nL/min. The full scan MS spectra ranged from 350 to 1300m/z and were acquired with a mass resolution of 70 K. Three biological replicates were used per sample.

MS/MS spectra were screened by Proteome Discoverer^TM^ 2.2 software (Thermo Fisher Scientific, United States) against the *uniprot-Acinetobacter johnsonii-taxonomy_40214-20190813, uniprot-Pseudomonas fluorescens-taxonomy_294-20190813* and the decoy database with following parameters. The highest score for a given peptide mass (best match to that predicted in the database) was used to identify parent proteins. The parameters for protein searching were set as follows: tryptic digestion with up to two missed cleavages, carbamidomethylation of cysteines as a fixed modification, and oxidation of methionines and protein *N*-terminal acetylation as variable modifications. A 1% false discovery rate (FDR) was used to identify peptide spectral matches based on *q*-Values.

### Statistical Analysis

All statistical values are expressed as the mean ± standard deviation. The biofilm formation, AHLs, AI-2 expression, and protease activity were analyzed by one-way analysis using SPSS 22.0 software (SPSS Inc., Chicago, IL, United States). The differential expression proteins were assigned to functional analysis according to the gene ontology (GO), clusters of orthologous groups of proteins (COG), and KEGG pathway 3 databases.

## Results and Discussion

### Identification and Detection of AHLs in *A. johnsonii*, *P. fluorescens*, and Co-culture

**FIGURE 1:**
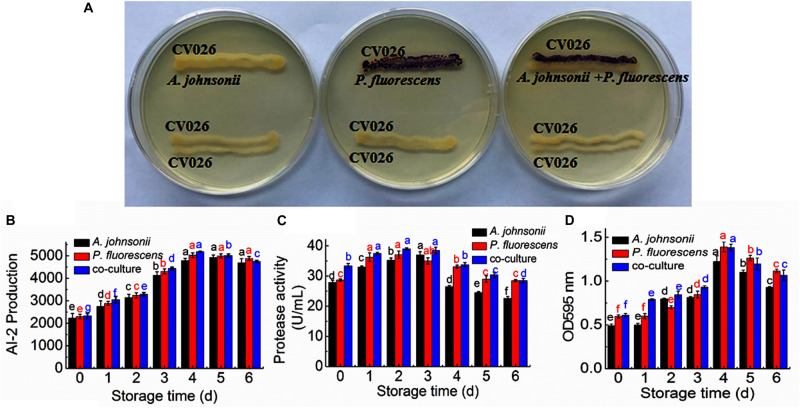
**(A)** Detection of AHL production in *A. johnsonii*, *P. fluorescens*, and their co-culture; changes in: **(B)** AI-2 production; **(C)** protease activity; and **(D)** biofilm production in *A. johnsonii*, *P. fluorescens*, and their co-culture after 6 days.

**FIGURE 2:**
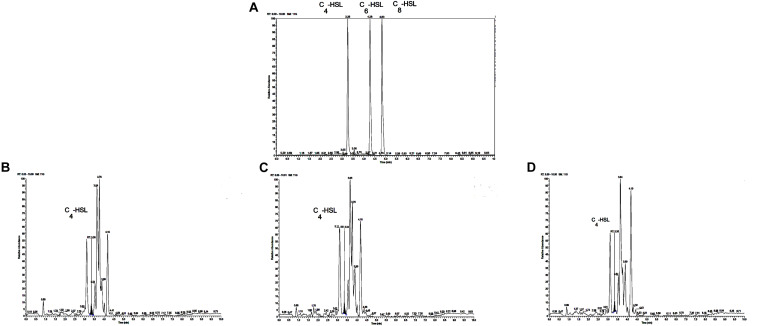
**(A)** Chromatograms of three kinds of AHL mixed standards. Chromatograms of C4-HSL produced by: **(B)** *A. johnsonii*; **(C)** *P. fluorescens*; and **(D)** their co-culture.

The reporter strain of the cross-feeding plate method is the fastest and most direct method to qualitatively detect AHLs in bacteria. CV026 is one of the commonly used reporter strains. CV026 does not produce AHLs by itself; however, CV026 is able to sense some of the AHLs of CivR protein (Yu et al., 2019). CV026 is fully capable of producing violacein in response to its cognate signal molecule. As shown in Figure 1A, *P. fluorescens* and the co-culture were capable of producing AHLs induced CV026 color reaction, while *A. johnsonii* could have low capable of producing AHLs not induce CV026 color reaction. The AHL signals for *A. johnsonii*, *P. fluorescens*, and their co-culture are shown in Figure 2 and Supplementary Figure S2. The three peaks with retention times of 3.26, 4.29, and 4.84 min were identified as C4-HSL, C6-HSL, and C8-HSL, respectively. The AHL (C4-HSL, C6-HSL, and C8-HSL) concentrations of the co-culture samples were significantly higher than those of *A. johnsonii* and *P. fluorescens*, indicating that the co-culture had pronounced AHL activities. The cooperative behaviors of the *A. johnsonii* and *P. fluorescens* cultures in response to AHLs activities might be to accelerate aquatic products spoilage. These trends are in agreement with the results presented in Table 1. The HPLC-MS chromatograms of the AHLs of the co-culture in C4-HSL, C6-HSL, and C8-HSL contained 3.145, 7.359, and 1.560 ng/ml, respectively (Table 1). Though several studies have reported that AHL signal molecules were the strongest autoinducers in Gram-negative bacteria from aquatic products (Zhu J. et al., 2015), reports of relative AHL production in *A. johnsonii* and its co-culture are limited.

**TABLE 1:** Contents of AHLs in *A. johnsonii*, *P. fluorescens* and co-culture.

**AHLs (ng/ml)**	***A. johnsonii***	***P. fluorescens***	**Co-culture**
C_4_-HSL	1.204 ± 0.076^c^	2.043 ± 0.060^b^	3.145 ± 0.014^a^
C_6_-HSL	7.325 ± 0.299^a^	6.802 ± 0.344^b^	7.359 ± 0.379^a^
C_8_-HSL	1.543 ± 0.110^a^	1.334 ± 0.308^b^	1.560 ± 0.554^a^

*^*a,b,c*^Means in the same line with different superscripts are significantly different (*p* < 0.05).*

### Detection of AI-2 Activity in *A. johnsonii*, *P. fluorescens*, and Co-culture

The activities of AI-2 signal molecules of **A. johnsonii**, **P. fluorescens**, and their co-culture were detected (Figure 1B). There were significant differences in the AI-2 activity of **A. johnsonii**, **P. fluorescens**, and their co-culture at different incubation times (**p** < 0.05). With increased culture time, the AI-2 activity first increased significantly from 0 to 4 days, then decreased, which was caused by increased bacterial growth density of bacteria, and bacteria in logarithmic phase. At the end of culture time, bacteria were in stationary phase and decline phase. Therefore, AI-2 activity decreased. It indicated that the changes in AI-2 activity were related to the secretion of the spoilage bacteria and environmental changes. Similar studies have shown that the AI-2 of QS is a global regulatory factor in aquatic products and influences the potential for spoilage (Peng et al., 2018; Li S. et al., 2019).

### Protease Activity of *A. johnsonii*, *P. fluorescens*, and Co-culture

Protease activity plays an essential role in food SSOs and is regulated by QS (Moradi et al., 2019; Li T. et al., 2020). Moreover, protease activity decomposes aquatic food proteins into small peptides and amino acids that are metabolized into volatile nitrogenous end products (Li J. et al., 2019). In this study, protease activity exhibited no significant effect after 3 days. The protease activity of *A. johnsonii*, *P. fluorescens*, and their co-culture first increased and then decreased (*p* < 0.05) during different culture periods (Figure 1C). The previous study reported that SSOs growth consumed low-molecular-weight compounds, and then protein was degraded by protease which caused SSOs growth and protease activity increase (Moradi et al., 2019). At the end of culture time, molecular-weight compounds were almost depleted. Therefore, protease activity decreased. The protease activities of the co-cultured samples were also higher than those of the single bacteria groups, in accord with the results reported in the literature (Li J. et al., 2019). This result suggests that protease activity as a key spoilage characteristic of co-cultured samples was regulated significantly, and at least partially, by an AHL-based QS system.

### Biofilm Formation by *A. johnsonii*, *P. fluorescens*, and Co-culture

Not only can biofilms influence food spoilage, resulting in reduced shelf life, but they can also adhere to bacteria and colonize surfaces (Zhou et al., 2019). Previous studies have provided some evidence to verify the correlation between biofilm formation and the SSOs of QS systems, by which exogenous bacteria can affect the formation of biofilms (Cui et al., 2020). Figure 1D shows that *A. johnsonii*, *P. fluorescens*, and their co-culture had the ability to form biofilms, which were greater in the co-cultured samples than those of the single bacteria. After 4d of culture, biofilm production reached maximum levels of 1.22, 1.38, and 1.39 by *A. johnsonii*, *P. fluorescens*, and their co-culture, respectively. Biofilm production increased when the incubation period was extended to 4d, but slowly decreased after 5d. Among the three groups, the presence of the co-cultured samples resulted in a significant increase in biofilm formation. This revealed that the cooperative behaviors of the *A. johnsonii* and *P. fluorescens* cultures in response to various signaling molecules helped to accelerate biofilm production.

### Proteome Analysis of *A. johnsonii*, *P. fluorescens*, and Co-culture

**FIGURE 3:**
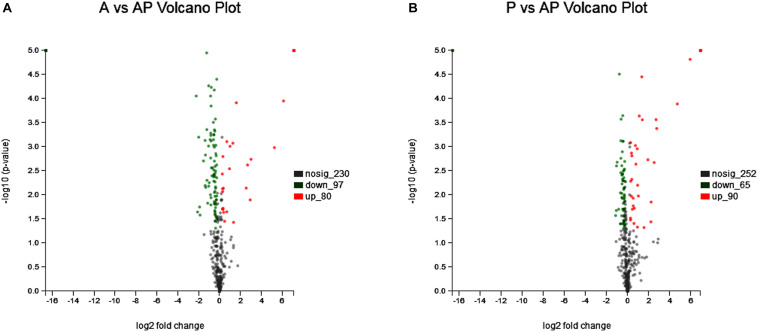
Volcanic map of all identified proteins: **(A)** volcanic map of all identified proteins in A vs. AP; **(B)** volcanic map of all identified proteins in P vs. AP. Red points: up-regulated proteins (fold change > 1.2, *p* < 0.05); green points: down-regulated proteins (fold change > 0.83, *p* < 0.05); black points: unchanged proteins.

*Acinetobacter johnsonii*, *P. fluorescens*, and their co-cultured samples were prepared using the label-free technique, which showed that a total of 1,176,121 spectra were identified in *A. johnsonii* and *P. fluorescens* by Proteome Discoverer^TM^ 2.2 software with a peptide FDR ≤ 0.01. As the fold change was >1.2 or <0.83, a *p*-value of <0.05 was used as the threshold to define the significance of the difference in protein expression. This enabled the quantification of 470 proteins and 444 proteins in *A. johnsonii* and *P. fluorescens*, respectively. As shown in Figure 3A, there were 80 significant up-regulated proteins and 97 down-regulated proteins in the A group, compared with the AP group. In addition, 90 up-regulated and 65 down-regulated proteins were in the P group, compared with the AP group (Figure 3B). The differences in protein expression are given in Tables 2, 3. Among the remarkably up-regulated proteins, the AI-2E family transporter OS, such as those of A0A2S8XHJ7, A0A166PHR0, A0A423M0X2, A0A2N1DUG2, and A0A165YHM6, showed notable up-regulation, which mediated the regulation of QS system expression (Quintieri et al., 2019; Saipriya et al., 2020). The result of AI-2 protein expression obtained above was similar to those shown in Figures 1B, 2. Moreover, some ribosomal proteins were significantly up-regulated, of which 50S ribosomal protein L3 OS, 50S ribosomal protein L25 OS, 30S ribosomal protein S17 OS, 50S ribosomal protein L23 OS, 30S ribosomal protein S2 OS, 50S ribosomal protein L29 OS, 50S ribosomal protein L10 OS, 50S ribosomal protein L4 OS, 30S ribosomal protein S10 OS, 30S ribosomal protein S4 OS, 50S ribosomal protein L9 OS, 50S ribosomal protein L15 OS, 50S ribosomal protein L11 OS, 50S ribosomal protein L6 OS, 50S ribosomal protein L7/L12 OS, 50S ribosomal protein L2 OS, and 50S ribosomal protein L1 OS were expressed at much higher levels, and their fold changes were all greater than 139.12. A similar result was tested for the progression of the ribosome through a regulatory open reading frame (ORF), which controlled the protein synthesis expression of many genes in *P. fluorescens* ITEM 17298 and influenced the expression of the downstream gene (Vazquez-Laslop et al., 2011; Gupta et al., 2013; Quintieri et al., 2019). The results showed that a majority of up-regulated proteins could accelerate protein expression and biological activity. There were some down-regulated proteins, such as thioredoxin reductase OS (A0A2K9M4Z2), cysteine synthase CysM OS (A0A506RJG5), DNA-binding response regulator (A0A423MKX5), amino acid ABC transporter ATPase OS (A0A0F4T6P4), that were expressed at a higher level in A vs. AP and P vs. AP. In the present study, bacteria played a critical role in transporting some molecules, including sugars, amino acids, vitamins, peptides, polysaccharides, lipids, thioredoxin, and ABC transporters (Rees et al., 2009; Zhong et al., 2019). Interestingly, more down-regulated proteins of the AI-2 family transporter OS were obtained in A vs. AP than in P vs. AP, which demonstrated more down-regulated proteins affecting AI-2 expression in *A. johnsonii*. Further investigations of AI-2 activity in *A. johnsonii* revealed it to be at a lower level than in the co-culture, suggesting that the latter might more easily contribute to AI-2 protein expression.

**TABLE 2:** The proteins differentially expression and most abundant proteins uniquely identified in A vs AP.

**Accession**	**Protein name**	**Regulate**	**Fold change**	***P*-value**
A0A0B7DGZ6	60 kDa chaperonin OS	Up	1.239	0.01994
A0A3S4MXA1	Chaperone protein HtpG OS	Up	1.287	0.007272
A0A3M4FN09	ATP-dependent protease ATPase subunit HslU OS	Up	1.223	0.007429
A0A4U3H0Y8	Thioredoxin TrxA OS	Up	1.225	0.008509
A0A166XFT9	RpoB (Fragment) OS	Up	1.309	0.02291
A0A4V5UF04	Molecular chaperone DnaK OS	Up	8.057	0.001808
A0A0D0SI76	Chaperone protein ClpB OS	Up	2.421	0.000832
A0A109KWR2	Chaperone protein HtpG OS	Up	38.01	0.001028
A0A3S4MHS8	ATP-dependent Clp protease ATP-binding subunit ClpA OS	Up	1.205	0.003655
A0A2W5E635	Elongation factor 4 OS	Up	1.213	0.01939
J2Y774	Thioredoxin reductase OS	Up	1.994	0.000966
A0A109KSE5	Arginine deiminase OS	Up	3.039	0.000112
A0A3M4FUT4	Carbamoyl-phosphate synthase large chain OS	Up	1.249	0.001582
A0A4R3X4W6	UDP-N-acetylglucosamine 2-epimerase OS	Up	1.269	0.019
A0A0D0SKK3	ATP synthase subunit alpha OS	Up	1.611	0.02238
A0A3M4FNE4	Elongation factor Tu OS	Up	69.56	0.000101
I4KE62	Ornithine carbamoyltransferase OS	Up	6.397	0.002375
A0A0D0PLG1	50S ribosomal protein L14 OS	Up	1.621	0.000769
Q3KIA0	Chaperone protein DnaK OS	Up	1.931	0.002845
A0A379IDI7	ABC transporter ATP-binding protein OS	Up	1.4	0.0352
A0A379IHY5	Transcriptional regulator MvaT, P16 subunit OS	Up	7.616	0.01267
A0A3M4G573	Dihydrolipoyllysine-residue succinyltransferase component of 2-oxoglutarate dehydrogenase complex OS	Up	2.525	0.03714
A0A0D0PNN7	Aldehyde dehydrogenase OS	Up	5.861	0.00719
U1TQN4	60 kDa chaperonin OS	Up	139.12	0
A0A4V5UFP6	Chaperonin GroEL OS	Up	139.12	0
A0A0B7DGY7	Elongation factor Tu OS	Up	139.12	0
A0A0W0HIU9	Alkyl hydroperoxide reductase C OS	Up	139.12	0
I4K6X1	Putative lipoprotein OS	Up	139.12	0
I4KG18	50S ribosomal protein L1 OS	Up	139.12	0
A0A109KXR2	Heat-shock protein OS	Up	139.12	0
A0A125QDK7	Elongation factor Ts OS	Up	139.12	0
A0A075PA24	10 kDa chaperonin OS	Up	139.12	0
A0A109KX60	Electron transfer flavoprotein subunit alpha OS	Up	139.12	0
A0A0X7K3A3	Dipicolinate synthase OS	Up	139.12	0
A0A010RMN0	Succinate–CoA ligase [ADP-forming] subunit alpha OS	Up	139.12	0
A0A4U3G4I4	Nucleotide exchange factor GrpE OS	Up	139.12	0
A0A0C2A4F3	Nucleoside diphosphate kinase OS	Up	139.12	0
A0A0A1YUR6	Membrane protein OS	Up	139.12	0
A0A1Q5X417	Cold-shock protein OS	Up	139.12	0
A0A0D0TC75	Succinate–CoA ligase [ADP-forming] subunit beta OS	Up	139.12	0
A0A0B7DIY4	50S ribosomal protein L2 OS	Up	139.12	0
A0A3M3XNL5	Ferritin domain-containing protein OS	Up	139.12	0
A0A0A1Z5I2	50S ribosomal protein L7/L12 OS	Up	139.12	0
A0A1T2YYC5	Ornithine aminotransferase OS	Up	139.12	0
A0A010SEN5	50S ribosomal protein L6 OS	Up	139.12	0
A0A010RGK5	Endoribonuclease OS	Up	139.12	0
A0A0A1YZ47	Transcriptional regulator HU subunit alpha OS	Up	139.12	0
A0A075P8Q2	30S ribosomal protein S4 OS	Up	139.12	0
A0A010SQL5	50S ribosomal protein L9 OS	Up	139.12	0
A0A0W0HKK7	50S ribosomal protein L15 OS	Up	139.12	0
A0A075PC10	50S ribosomal protein L11 OS	Up	139.12	0
A0A0C1ZKZ7	Lipoprotein OS	Up	139.12	0
A0A010RRM4	30S ribosomal protein S10 OS	Up	139.12	0
C3K254	Osmotically inducible protein Y OS	Up	139.12	0
A0A0A1Z8J0	50S ribosomal protein L4 OS	Up	139.12	0
A0A3M5MJ63	Fatty acid oxidation complex subunit alpha OS	Up	139.12	0
A0A0N7H007	Urocanate hydratase OS	Up	139.12	0
E2XZ08	50S ribosomal protein L29 OS	Up	139.12	0
A0A0W0HLG0	50S ribosomal protein L10 OS	Up	139.12	0
A0A3M3Y045	Aspartate ammonia-lyase OS	Up	139.12	0
A0A448BWD3	Spermidine/putrescine import ATP-binding protein PotA OS	Up	139.12	0
A0A3M3XD24	Nucleoid-associated protein ALQ35_00435 OS	Up	139.12	0
A0A0W0HH67	Chromosome partitioning protein ParA OS	Up	139.12	0
A0A010RSX6	Adenosylhomocysteinase OS	Up	139.12	0
A0A120G5R9	DUF2383 domain-containing protein OS	Up	139.12	0
A0A109KMT8	2,3,4,5-tetrahydropyridine-2,6-dicarboxylate N-succinyltransferase OS	Up	139.12	0
A0A0G4E5Q2	Single-stranded DNA-binding protein OS	Up	139.12	0
A0A0K1QL49	Protein-export protein SecB OS	Up	139.12	0
A0A109KQK7	Uncharacterized protein OS	Up	139.12	0
A0A3M3XER7	50S ribosomal protein L3 OS	Up	139.12	0
A0A387BYY7	50S ribosomal protein L25 OS	Up	139.12	0
U1TYF4	30S ribosomal protein S17 OS	Up	139.12	0
A0A3M3XF13	50S ribosomal protein L23 OS	Up	139.12	0
A0A3M5N9X5	30S ribosomal protein S2 OS	Up	139.12	0
A0A3M4G1L6	Aldedh domain-containing protein OS	Up	139.12	0
A0A0W0H1J6	Elongation factor P OS	Up	139.12	0
A0A3M5N1D0	Superoxide dismutase OS	Up	139.12	0
A0A0D0TBX7	OmpW protein OS	Up	139.12	0
A0A109LGM0	Cysteine synthase B OS	Up	139.12	0
A0A2S8XHJ7	AI-2E family transporter OS	Up	139.12	0
A0A109LMS3	Cupin domain protein OS	Down	0.7153	0.000434
A0A0B7DGC9	Serine hydroxymethyltransferase OS	Down	0.78	0.001373
A0A3S4N2A1	DNA-binding transcriptional dual regulator Crp OS	Down	0.7301	0.000463
A0A3M5MWI0	ATP-dependent Clp protease ATP-binding subunit ClpX OS	Down	0.8297	0.00003
A0A3M4GP41	Threonine–tRNA ligase OS	Down	0.7987	0.001523
A0A0C1X1G2	Ribosomal RNA large subunit methyltransferase J OS	Down	0.8124	0.02763
A0A010SIG9	Aconitate hydratase OS	Down	0.6839	0.000558
A0A3S4MM74	2-dehydro-3-deoxyphosphooctonate aldolase OS	Down	0.7282	0.02122
A0A263S7G8	Elongation factor Tu (Fragment) OS	Down	0.2323	0.0226
A0A448BG48	4-hydroxy-3-methylbut-2-en-1-yl diphosphate synthase (flavodoxin) OS	Down	0.5716	0.000746
A0A3S4RD08	Glycerol kinase OS	Down	0.7652	0.01836
A0A448BUH7	Transcription termination factor Rho OS	Down	0.7681	0.04901
A0A109LM37	DUF86 domain-containing protein OS	Down	0.7598	0.000255
A0A3S4N8H0	Pyruvate kinase OS	Down	0.5626	0.01609
A0A3M3XN23	Adenylosuccinate lyase OS	Down	0.8096	0.01257
A0A161Z4S7	Glycerol-3-phosphate dehydrogenase OS	Down	0.5523	0.000078
A0A448BM26	Elongation factor G OS	Down	0.679	0.003014
A0A4R3X0P0	Integration host factor subunit alpha OS	Down	0.5545	0.001699
A0A3S4SXK6	Transcription termination/antitermination protein NusG OS	Down	0.7081	0.001008
A0A0K1QXR7	Glycerol kinase OS	Down	0.7005	0.0138
A0A345V0J2	dTDP-4-dehydrorhamnose reductase OS	Down	0.5598	0.000511
G8Q3T2	Pyruvate dehydrogenase E1 component OS	Down	0.781	0.00092
E2XZ43	Transcriptional regulator, Crp/Fnr family OS	Down	0.7111	0.001972
A0A109LMK0	Nucleotidyltransferase domain protein OS	Down	0.627	0.002719
A0A1T2YJX3	Glycerol kinase OS	Down	0.4863	0.006571
A0A4R3XCE2	Uncharacterized protein OS	Down	0.7417	0.00254
A0A010RT21	Site-determining protein OS	Down	0.5787	0.002727
A0A0D9AUP5	60 kDa chaperonin OS	Down	0.2131	0.000078
A0A448BVJ5	ATP synthase subunit alpha OS	Down	0.6884	0.003857
A0A0B7D324	Translation initiation factor IF-2 OS	Down	0.7328	0.02568
A0A010S2I6	GTP-binding protein TypA OS	Down	0.4553	0.0148
A0A3S4MJG8	Succinate–CoA ligase [ADP-forming] subunit beta OS	Down	0.717	0.01519
A0A109L0L2	Uncharacterized protein OS	Down	0.4868	0.000044
A0A3S4MGB5	NAD-dependent malic enzyme OS	Down	0.826	0.002371
A0A0F4VFT8	Quinone oxidoreductase OS	Down	0.6851	0.000545
A0A3M5MJB2	DNA topoisomerase 1 OS	Down	0.826	0.01052
A0A3M4G5X7	Alanine–tRNA ligase OS	Down	0.8054	0.01262
A0A448BUH9	Glycine dehydrogenase (decarboxylating) OS	Down	0.6628	0.0003
A0A3M3XUU9	Glucans biosynthesis glucosyltransferase H OS	Down	0.43	0.000429
A0A370XHT9	G/U mismatch-specific DNA glycosylase OS	Down	0.7478	0.003514
A0A109KNB3	Glutathione peroxidase OS	Down	0.6022	0.003997
A0A0F4TQL9	Sulfate adenylyltransferase subunit 2 OS	Down	0.5683	0.000132
A0A448BV68	Protease OS	Down	0.7601	0.01423
A0A3M3XL87	Carbonic anhydrase OS	Down	0.5356	0.000698
A0A3S4MT74	2Fe-2S ferredoxin OS	Down	0.6699	0.000918
A0A010SYA6	UDP-N-acetylmuramate–L-alanine ligase OS	Down	0.7554	0.03542
A0A0D0TN17	NAD/NADP-dependent betaine aldehyde dehydrogenase OS	Down	0.7868	0.02845
A0A3S4PY88	Succinate–CoA ligase [ADP-forming] subunit alpha OS	Down	0.753	0.0247
A0A1T2Y0Q8	Oxidoreductase OS	Down	0.7698	0.00724
E2XYQ5	UDP-N-acetylmuramate–L-alanyl-gamma-D-glutamyl-meso-2,6-diaminoheptandioate ligase OS	Down	0.7967	0.01584
A0A3M4G016	Lipoyl synthase OS	Down	0.7383	0.01066
A0A0D9B4C3	NADH-quinone oxidoreductase subunit I OS	Down	0.7234	0.01535
A0A0F4TK66	Dienelactone hydrolase OS	Down	0.6862	0.003742
A0A010RIC6	Multifunctional fusion protein OS	Down	0.4251	0.000001
A0A3S4PCY6	NADH-quinone oxidoreductase subunit F OS	Down	0.8283	0.004383
A0A263S827	UDP-glucose 6-dehydrogenase (Fragment) OS	Down	0.7358	0.008413
A0A010TH85	Glucarate dehydratase OS	Down	0.7664	0.003172
B3PL47	Thioredoxin reductase OS	Down	0.3985	0.006945
A0A010T8W6	Succinate dehydrogenase flavoprotein subunit OS	Down	0.7114	0.008916
A0A379J8W9	Alkyl hydroperoxide reductase OS	Down	0.4814	0.000953
A0A0X7K8K2	Alkene reductase OS	Down	0.7332	0.004101
L7H6Z5	1,4-alpha-glucan branching enzyme GlgB OS	Down	0.7367	0.005334
A0A0B7DGX9	30S ribosomal protein S7 OS	Down	0.3928	0.001471
A0A3M4FP51	Bifunctional protein PutA OS	Down	0.5788	0.000048
A0A0A1YRY8	Peroxiredoxin OsmC OS	Down	0.6682	0.000699
A0A0D0NJK9	NADH-quinone oxidoreductase subunit C/D OS	Down	0.5453	0.009922
A0A0F4T6P4	Amino acid ABC transporter ATPase OS	Down	0.2658	0.01775
A0A423M0U6	Microcin ABC transporter ATP-binding protein OS	Down	0.6836	0.005146
A0A379IA35	Cysteine synthase OS	Down	0.5603	0.00057
A0A120G9E8	DNA-invertase hin OS	Down	0.5688	0.0114
A0A3S4RLS6	50S ribosomal protein L17 OS	Down	0.5046	0.00556
A0A2A5REX4	AI-2E family transporter	Down	0.7659	0.006788
A0A3M3XCL9	Phosphoenolpyruvate synthase OS	Down	0.2506	0.000621
A0A010SRH4	Phosphoribosylglycinamide formyltransferase OS	Down	0.7039	0.000056
I4K120	Malonate decarboxylase, gamma subunit OS	Down	0.7353	0.02236
A0A0C1WLI8	30S ribosomal protein S13 OS	Down	0.3449	0.001954
A0A3M3XP35	Aldedh domain-containing protein OS	Down	0.3826	0.000729
A0A1B3D5W7	AI-2E family transporter OS	Down	0.7473	0.03427
A0A2N1DZF0	Type VI secretion system tube protein Hcp OS	Down	0.3845	0.006341
A0A4P7I7D8	AAA family ATPase OS	Down	0.4335	0.004957
A0A3M4GSW6	DNA polymerase III subunit alpha OS	Down	0.6754	0.004196
A0A0P8X0Z8	Succinate dehydrogenase and fumarate reductase iron-sulfur family protein OS	Down	0.2746	0.02616
A0A1T2ZCZ1	Alkyl hydroperoxide reductase C OS	Down	0.00001	0
A0A109KFA5	Peptide methionine sulfoxide reductase MsrA OS	Down	0.00001	0
A0A0W0HVB3	Electron transfer flavoprotein subunit beta OS	Down	0.00001	0
A0A423N111	TetR family transcriptional regulator OS	Down	0.00001	0
A0A2W5EAC0	Citrate synthase OS	Down	0.00001	0
A0A109FQR8	Ketol-acid reductoisomerase (NADP(+)) OS	Down	0.00001	0
A0A423MKX5	DNA-binding response regulator OS	Down	0.00001	0
A0A0B7D391	1-(5-phosphoribosyl)-5-[(5-phosphoribosylamino) methylideneamino] imidazole-4-carboxamide isomerase OS	Down	0.00001	0
A0A0B7DDH5	Iron-sulfur cluster assembly scaffold protein IscU OS	Down	0.00001	0
A0A327NA26	AI-2E family transporter OS	Down	0.00001	0
A0A327MWL2	AI-2E family transporter OS	Down	0.00001	0
A0A2K9M4Z2	Thioredoxin reductase OS	Down	0.00001	0
A0A506RJG5	Cysteine synthase CysM OS	Down	0.00001	0
A0A423L5V9	AI-2E family transporter OS	Down	0.00001	0
A0A2A5R442	AI-2E family transporter OS	Down	0.00001	0

**TABLE 3:** The proteins differentially expression and most abundant proteins uniquely identified in P vs. AP.

**Accession**	**Protein name**	**Regulate**	**Fold change**	***p*-value**
A0A448DVQ8	Thioredoxin reductase OS	Up	1.226	0.005186
A0A010S5P5	Electron transfer flavoprotein subunit beta OS	Up	1.519	0.03911
A0A109KZR3	30S ribosomal protein S1 OS	Up	1.22	0.000809
A0A3S4NMX4	Cysteine synthase OS	Up	1.447	0.01686
A0A010T165	Cysteine desulfurase IscS OS	Up	1.316	0.004729
A0A166PHR0	AI-2E family transporter OS	Up	1.441	0.01166
A0A3S4NMW4	Proline–tRNA ligase OS	Up	1.213	0.03312
A0A423M0X2	AI-2E family transporter OS	Up	1.21	0.03034
A0A423LFX7	Glycine betaine ABC transporter substrate-binding protein OS	Up	1.929	0.006274
A0A3M4HEE6	Fn3_like domain-containing protein OS	Up	1.361	0.01977
A0A109L3N2	Elongation factor G OS	Up	1.288	0.001337
A0A0D0SI76	Chaperone protein ClpB OS	Up	2.025	0.0105
A0A4V5UF04	Molecular chaperone DnaK OS	Up	6.781	0.000406
A0A0B7DHH8	S-adenosylmethionine:tRNA ribosyltransferase-isomerase OS	Up	1.319	0.001532
A0A109KSE5	Arginine deiminase OS	Up	2.538	0.000025
A0A109KWR2	Chaperone protein HtpG OS	Up	26.25	0.000119
A0A3M4FNE4	Elongation factor Tu OS	Up	61.14	0.000005
I4KE62	Ornithine carbamoyltransferase OS	Up	5.734	0.002111
A0A0X7K650	Alkyl hydroperoxide reductase OS	Up	2.631	0.000265
I4K7P9	ATP synthase subunit beta OS	Up	4.626	0.03624
A0A0C1WLI8	30S ribosomal protein S13 OS	Up	2.938	0.04752
A0A3M4GZQ4	Ribose-phosphate pyrophosphokinase OS	Up	1.725	0.00228
A0A0D0PLG1	50S ribosomal protein L14 OS	Up	1.668	0.000929
A0A010RPF8	16S rRNA methyltransferase OS	Up	1.548	0.01886
A0A3M4G573	Dihydrolipoyllysine-residue succinyltransferase component of 2-oxoglutarate dehydrogenase complex OS	Up	4.699	0.01403
A0A379IDI7	ABC transporter ATP-binding protein OS	Up	1.278	0.01057
A0A2K9M4Z2	Thioredoxin reductase OS	Up	1.908	0.04652
A0A2N1DUG2	AI-2E family transporter OS	Up	2.147	0.000219
A0A379IHY5	Transcriptional regulator MvaT, P16 subunit OS	Up	6.47	0.000263
A0A165YHM6	AI-2E family transporter OS	Up	1.881	0.00109
A0A0D0PNN7	Aldehyde dehydrogenase OS	Up	3.797	0.001845
U1TQN4	60 kDa chaperonin OS	Up	122.28	0
A0A4V5UFP6	Chaperonin GroEL OS	Up	122.28	0
A0A0W0HIU9	Alkyl hydroperoxide reductase C OS	Up	122.28	0
I4K6X1	Putative lipoprotein OS	Up	122.28	0
I4KG18	50S ribosomal protein L1 OS	Up	122.28	0
A0A109KXR2	Heat-shock protein OS	Up	122.28	0
A0A125QDK7	Elongation factor Ts OS	Up	122.28	0
A0A075PA24	10 kDa chaperonin OS	Up	122.28	0
A0A109KX60	Electron transfer flavoprotein subunit alpha OS	Up	122.28	0
A0A0X7K3A3	Dipicolinate synthase OS	Up	122.28	0
A0A010RMN0	Succinate–CoA ligase [ADP-forming] subunit alpha OS	Up	122.28	0
A0A4U3G4I4	Nucleotide exchange factor GrpE OS	Up	122.28	0
A0A0C2A4F3	Nucleoside diphosphate kinase OS	Up	122.28	0
A0A0A1YUR6	Membrane protein OS	Up	122.28	0
A0A1Q5X417	Cold-shock protein OS	Up	122.28	0
A0A0D0TC75	Succinate–CoA ligase [ADP-forming] subunit beta OS	Up	122.28	0
A0A0B7DIY4	50S ribosomal protein L2 OS	Up	122.28	0
A0A0D0PJB7	Enolase OS	Up	122.28	0
A0A3M3XNL5	Ferritin domain-containing protein OS	Up	122.28	0
A0A0A1Z5I2	50S ribosomal protein L7/L12 OS	Up	122.28	0
A0A1T2YYC5	Ornithine aminotransferase OS	Up	122.28	0
A0A010SEN5	50S ribosomal protein L6 OS	Up	122.28	0
A0A010RGK5	Endoribonuclease OS	Up	122.28	0
A0A0A1YZ47	Transcriptional regulator HU subunit alpha OS	Up	122.28	0
A0A075P8Q2	30S ribosomal protein S4 OS	Up	122.28	0
A0A010SQL5	50S ribosomal protein L9 OS	Up	122.28	0
A0A0W0HKK7	50S ribosomal protein L15 OS	Up	122.28	0
A0A075PC10	50S ribosomal protein L11 OS	Up	122.28	0
A0A0C1ZKZ7	Lipoprotein OS	Up	122.28	0
A0A010RRM4	30S ribosomal protein S10 OS	Up	122.28	0
C3K254	Osmotically inducible protein Y OS	Up	122.28	0
A0A0A1Z8J0	50S ribosomal protein L4 OS	Up	122.28	0
A0A3M5MJ63	Fatty acid oxidation complex subunit alpha OS	Up	122.28	0
A0A0N7H007	Urocanate hydratase OS	Up	122.28	0
E2XZ08	50S ribosomal protein L29 OS	Up	122.28	0
A0A0W0HLG0	50S ribosomal protein L10 OS	Up	122.28	0
A0A448BQI1	Phosphoribosylformylglycinamidine cyclo-ligase OS	Up	122.28	0
A0A3M3Y045	Aspartate ammonia-lyase OS	Up	122.28	0
A0A448BWD3	Spermidine/putrescine import ATP-binding protein PotA OS	Up	122.28	0
A0A0W0QVH5	Porin OS	Up	122.28	0
A0A3M3XD24	Nucleoid-associated protein ALQ35_00435 OS	Up	122.28	0
A0A3M4GIL8	Fumarate hydratase class I OS	Up	122.28	0
A0A0W0HH67	Chromosome partitioning protein ParA OS	Up	122.28	0
A0A010RSX6	Adenosylhomocysteinase OS	Up	122.28	0
A0A0K1QHC1	50S ribosomal protein L18 OS	Up	122.28	0
A0A109KMT8	2,3,4,5-tetrahydropyridine-2,6-dicarboxylate N-succinyltransferase OS	Up	122.28	0
A0A0G4E5Q2	Single-stranded DNA-binding protein OS	Up	122.28	0
A0A109KQK7	Uncharacterized protein OS	Up	122.28	0
A0A3M3XER7	50S ribosomal protein L3 OS	Up	122.28	0
A0A387BYY7	50S ribosomal protein L25 OS	Up	122.28	0
U1TYF4	30S ribosomal protein S17 OS	Up	122.28	0
A0A3M3XF13	50S ribosomal protein L23 OS	Up	122.28	0
A0A3M5N9X5	30S ribosomal protein S2 OS	Up	122.28	0
A0A3S4RR46	Nitrogen regulatory protein PII OS	Up	122.28	0
A0A3M5MIC6	S1 motif domain-containing protein OS	Up	122.28	0
A0A3M4G1L6	Aldedh domain-containing protein OS	Up	122.28	0
A0A0W0H1J6	Elongation factor P OS	Up	122.28	0
A0A3M5N1D0	Superoxide dismutase OS	Up	122.28	0
A0A2S8XHJ7	AI-2E family transporter OS	Up	122.28	0
A0A109LMS3	Cupin domain protein OS	Down	0.7207	0.003665
A0A0B7DGC9	Serine hydroxymethyltransferase OS	Down	0.706	0.001258
A0A3M5MWI0	ATP-dependent Clp protease ATP-binding subunit ClpX OS	Down	0.798	0.00304
A0A010T1X2	50S ribosomal protein L5 OS	Down	0.7538	0.005059
A0A448BG48	4-hydroxy-3-methylbut-2-en-1-yl diphosphate synthase (flavodoxin) OS	Down	0.6935	0.005875
A0A109LM37	DUF86 domain-containing protein OS	Down	0.7715	0.007694
A0A3S4N8H0	Pyruvate kinase OS	Down	0.616	0.03967
A0A0A1ZAA7	Glucose-1-phosphate thymidylyltransferase OS	Down	0.7914	0.02799
A0A161Z4S7	Glycerol-3-phosphate dehydrogenase OS	Down	0.7438	0.003224
A0A3M3XN23	Adenylosuccinate lyase OS	Down	0.8242	0.005162
A0A448BGH7	Phosphoenolpyruvate carboxylase OS	Down	0.7643	0.01978
A0A3S4SXK6	Transcription termination/antitermination protein NusG OS	Down	0.7793	0.03637
A0A345V0J2	dTDP-4-dehydrorhamnose reductase OS	Down	0.6827	0.002461
E2XZ43	Transcriptional regulator, Crp/Fnr family OS	Down	0.8076	0.01046
A0A010RT21	Site-determining protein OS	Down	0.7867	0.01215
A0A109LMK0	Nucleotidyltransferase domain protein OS	Down	0.7134	0.003733
A0A4R3XCE2	Uncharacterized protein OS	Down	0.8129	0.0114
A0A0N9VVZ3	Phosphoserine aminotransferase OS	Down	0.8173	0.04239
A0A3S4N6L6	Isocitrate dehydrogenase [NADP] OS	Down	0.78	0.02703
A0A379J9N3	Thioredoxin reductase OS	Down	0.7602	0.02114
A0A4Y4JC04	Cysteine synthase B OS	Down	0.8055	0.007142
A0A3S4MJG8	Succinate–CoA ligase [ADP-forming] subunit beta OS	Down	0.7899	0.0351
A0A3M5MSW3	UvrABC system protein B OS	Down	0.8096	0.002896
A0A109L0L2	Uncharacterized protein OS	Down	0.6859	0.003862
A0A379J4U0	Cysteine synthase OS	Down	0.7646	0.03365
A0A0A1YZP1	Histidine–tRNA ligase OS	Down	0.7848	0.00437
A0A3M4G5X7	Alanine–tRNA ligase OS	Down	0.7532	0.01493
A0A0K1QSD3	LysR family transcriptional regulator OS	Down	0.8259	0.04135
A0A370XHT9	G/U mismatch-specific DNA glycosylase OS	Down	0.6979	0.01167
A0A3M3XL87	Carbonic anhydrase OS	Down	0.68	0.009262
A0A448BV68	Protease OS	Down	0.8028	0.01425
A0A109KNB3	Glutathione peroxidase OS	Down	0.4815	0.002483
A0A3S4MT74	2Fe-2S ferredoxin OS	Down	0.7981	0.01977
A0A010RGL6	Phosphoglucomutase OS	Down	0.7412	0.000758
Q4K9V2	Thioredoxin reductase OS	Down	0.7618	0.03801
A0A3M4G016	Lipoyl synthase OS	Down	0.7969	0.03549
A0A010RIC6	Multifunctional fusion protein OS	Down	0.5799	0.000021
A0A0D9B4C3	NADH-quinone oxidoreductase subunit I OS	Down	0.64	0.00074
A0A010RSQ5	L-cystine transporter tcyP OS	Down	0.5553	0.01979
A0A0F4T9J8	Diaminopimelate decarboxylase OS	Down	0.8255	0.002003
B3PL47	Thioredoxin reductase OS	Down	0.5017	0.002077
A0A010T8W6	Succinate dehydrogenase flavoprotein subunit OS	Down	0.8224	0.04234
A0A0B7DI73	Isoleucine–tRNA ligase OS	Down	0.7257	0.000215
A0A3M4GK93	Phosphoribosylaminoimidazole-succinocarboxamide synthase OS	Down	0.686	0.01121
A0A3M5MVH0	Glucans biosynthesis protein D OS	Down	0.6468	0.03983
A0A0X7K8K2	Alkene reductase OS	Down	0.8053	0.00987
A0A3M4FP51	Bifunctional protein PutA OS	Down	0.6509	0.000256
A0A0A1YRY8	Peroxiredoxin OsmC OS	Down	0.7261	0.005055
A0A3S4N1Z0	Cytosine deaminase OS	Down	0.7876	0.007186
A0A0N9W7I2	Catalase OS	Down	0.6428	0.001721
A0A0F4T6P4	Amino acid ABC transporter ATPase OS	Down	0.474	0.02104
A0A0B7D1W3	Glycine betaine transport ATP-binding protein OpuAA OS	Down	0.7214	0.01965
A0A379IA35	Cysteine synthase OS	Down	0.7158	0.004647
I4K120	Malonate decarboxylase, gamma subunit OS	Down	0.8073	0.01994
A0A010SRH4	Phosphoribosylglycinamide formyltransferase OS	Down	0.7332	0.000756
A0A327MWL2	AI-2E family transporter OS	Down	0.6227	0.009033
A0A0N8NY49	Aldo/keto reductase family protein OS	Down	0.7843	0.04723
A0A0B7D6X0	Glutathione import ATP-binding protein GsiA OS	Down	0.6366	0.003439
A0A0F4TUQ5	Glucose dehydrogenase OS	Down	0.454	0.02673
A0A0P9BDW1	Ribose import ATP-binding protein RbsA OS	Down	0.6737	0.02709
A0A3S4PXV5	Transcriptional activator CopR OS	Down	0.731	0.03974
A0A423N111	TetR family transcriptional regulator OS	Down	0.00001	0
A0A0B7DDH5	Iron-sulfur cluster assembly scaffold protein IscU OS	Down	0.00001	0
A0A125QFU4	AI-2E family transporter OS	Down	0.00001	0
A0A386Y9V3	AI-2E family transporter OS	Down	0.00001	0

### Bioinformatics Functional Analysis of *A. johnsonii*, *P. fluorescens*, and Co-culture

#### GO and COG Enrichment Analysis of *A. johnsonii*, *P. fluorescens*, and Co-culture

**FIGURE 4:**
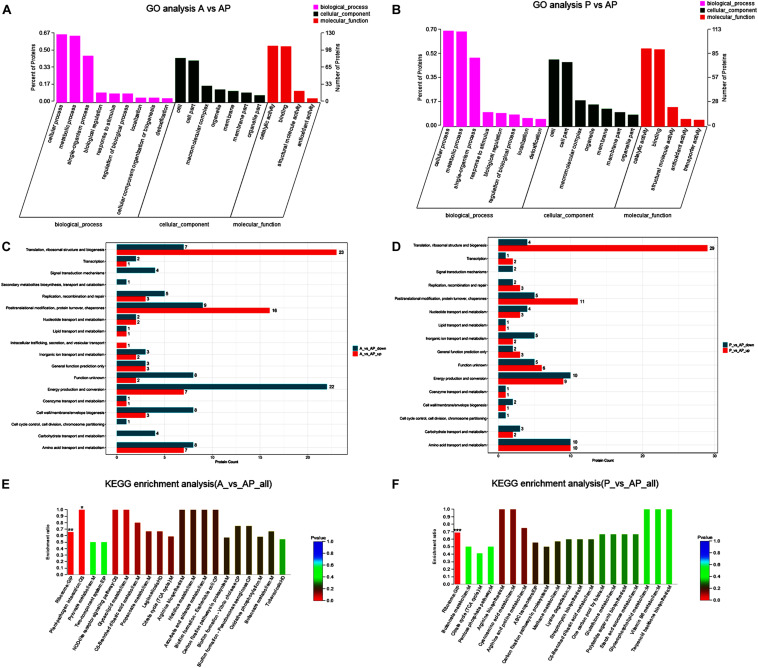
Gene ontology terms of the differentially expressed proteins in: **(A)** A vs. AP; **(B)** P vs. AP. COG terms of the differentially expressed proteins in: **(C)** A vs. AP; **(D)**: P vs. AP. KEGG pathway analysis of the differentially expressed proteins in: **(E)** A vs. AP; **(F)** P vs. AP. The red bars represented the up-regulated proteins, and the blue bars represent the down-regulated proteins in the KEGG pathway analysis of *A. johnsonii*, *P. fluorescens*, and their co-culture. *Candida albicans* glycolysis/gluconeogenesis pathways **(A)** and peroxisomal assembly and fatty acid oxidation pathways.

A GO classification and COG enrichment of the 177 and 155 differentially expressed proteins were performed in A vs. AP and P vs. AP, respectively (Figures 4A,B). There were three main categories of cellular components, biological processes, and molecular functions in the GO classification. The cellular process (GO:0009987), the metabolic process (GO:0008152), and the single-organism process (GO:0044699) were the three main distributed terms in the biological processes. The 85 and 79 differentially expressed proteins were annotated as belonging to the cell in A vs. AP and P vs. AP, respectively. In addition, A vs. AP (80 out of 177) and P vs. AP (76 out of 155) of the differentially expressed proteins were localized in the cell part. This suggested that *A. johnsonii*, *P. fluorescens*, and their co-culture played an essential role in the transmembrane transport function and intracellular and extracellular substance migration, thereby promoting nutrient absorption and excretion of metabolic products. In the molecular functions category, the proteins were related to catalytic activity and binding.

Figures 4C,D displays the COG enrichment analysis. A total of 18 categories were classified, in which the top 6 COG terms were (i) energy production and conversion, (ii) amino acid transport and metabolism, (iii) translation, ribosomal structure, and biogenesis, (iv) post-translational modification, (v) protein turnover, and (vi) chaperones. A total of 22 down-regulated proteins were involved in energy production and conversion in A vs. AP, while 23 up-regulated proteins were involved in translation, ribosomal structure, and biogenesis in A vs. AP. Interestingly, translation, ribosomal structure, and biogenesis were significantly up-regulated in P vs. AP, and amino acid transport and metabolism and energy production and conversion were significantly down-regulated in P vs. AP, suggesting that translation, ribosomal structure, and energy production and conversion were valuable targets for co-culturing, and thus deserve further investigation. It has been suggested that under co-culture conditions, energy production is capable of involving translation of the bacteria, resulting in the activation of the ribosomal structure (Gupta et al., 2013). These proteins might also participate in nucleotide catabolism, allowing bacteria to use deoxynucleotides as energy sources. The results of GO analysis and COG enrichment provide a significant view of the proteins differentially expressed in *A. johnsonii*, *P. fluorescens*, and their co-culture that can elevate protein functions.

#### Pathway Enrichment Analysis of *A. johnsonii*, *P. fluorescens*, and Co-culture

Pathway enrichment analysis of the KEGG database was conducted on the differentially expressed proteins to reveal the metabolic and signal transduction pathways of *A. johnsonii*, *P. fluorescens*, and their co-culture (Figures 4E,F). In our study, taken together, the up/down-regulated differentially expressed proteins of *A. johnsonii*, *P. fluorescens*, and their co-culture involved in amino acid metabolism, carbohydrate and energy metabolism, and nucleotide metabolism enabled predictions of the change in the culture of the *A. johnsonii*, *P. fluorescens* samples in the co-culture, indicating that protein changes and carbohydrate transformation contributed to bacteria co-culturing. A total of 84 and 77 differentially expressed proteins in A vs. AP and P vs. AP, respectively, were divided into 20 KEGG pathways, and a majority of the metabolic pathways included genetic information processing, environmental information processing, cellular processes, organismal systems, and human diseases. The KEGG pathways of amino acid metabolism, carbohydrate metabolism, energy metabolism, and translation were significantly enriched. A similar result for carbohydrate metabolism and energy metabolism of *Vibrio parahaemolyticus* revealed that carbohydrate metabolism is the key factor, indicating that carbohydrate can either be converted into glucan and fructose through a glycosyltransferase reaction or transported by sugar transport systems and subsequently metabolized through glycolysis (Zhong et al., 2019).

The main significant items relevant to the regulation of biofilm formation of A vs. AP and P vs. AP included the map 02026, map 05111, and map 02025 pathways (Supplementary Tables S1, S2). Analysis of the proteins of *A. johnsonii*, *P. fluorescens*, and their co-culture indicated that all of the pathways were present. In addition, the pathway of map 02024 was that which regulated QS, which is a crucial feature affecting the regulation of biofilm formation by AHLs, bacterial growth, protease activity, and the spoilage potential of bacteria (Jie et al., 2018).

Furthermore, as shown in Supplementary Tables S1, S2, ribosome was the most significantly enriched pathway, which indicated that protein expression was substantially promoted to achieve a large demand for bacterial growth (Li J. et al., 2020). Ribosome was the most significant pathway, with 43 differentially expressed proteins, of which 22 were up-regulated proteins and 21 were down-regulated proteins. In addition, abundant proteins were associated with the tricarboxylic acid (TCA) cycle and oxidative phosphorylation in response to co-culture conditions (Supplementary Figure S1). The TCA cycle pathway has 7 up-regulated proteins and 9 down-regulated proteins. The most abundant protein detected among the 6 up-regulated and 11 down-regulated proteins was associated with oxidative phosphorylation. These pathways were essential for bacterial growth and cell interactions, which have potential for enhancing bacterial spoilage and QS regulation (Remenant et al., 2015; Otwell et al., 2018).

### Subcellular Localization Prediction of *A. johnsonii*, *P. fluorescens*, and Co-culture

**FIGURE 5:**
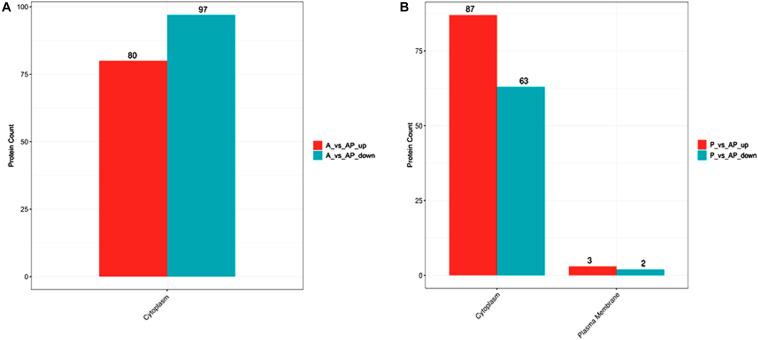
Bar plot of subcellular localization prediction in: **(A)** A vs. AP; **(B)** P vs. AP.

The subcellular localization prediction of proteins has attracted considerable attention in protein functional annotation. There were two subcellular localization predictions, including for cytoplasm and plasma membranes. Figure 5 shows that subcellular localization in P vs. AP was located mainly in the cytoplasm and the plasma membrane, but mainly in the cytoplasm in A vs. AP. Previous studies have focused mainly on single bacteria producing a few metabolites that migrate from the inner membrane to cell-extracellular membranes during culture (Ellepola et al., 2019), resulting in cell communications. However, few studies have reported explanations for two bacterial subcellular localization predictions. As shown in Figure 5, expression of cytoplasmic proteins was also regulated in the co-culture.

### Quorum Sensing and Spoilage-Related Proteins

The QS mechanism is the means by which cell population communication can regulate specific proteins to express the physiological characteristics of microorganisms (Li S. et al., 2019). As given in Table 4, the QS system was related to the protein of the AI-2E family transporter OS and the LuxR family transcriptional regulator OS. The LuxR family transcriptional regulator was found in A vs. AP and P vs. AP. The LuxR protein (AHL receptor) commonly consists of 200–260 amino acids blinding with the key protein, resulting in the expression of the key protein. Five AI-2E family transporter up-regulated proteins and 11 AI-2E family transporter up-regulated proteins were found in A vs. AP and P vs. AP, respectively. This suggests that the AI-2E family transporter played a vital role in regulating QS. Previous studies have shown that *P. fluorescens* can produce AI-2 proteins and AHLs (Sharma et al., 2006), which is consistent with the results described in Section “Protease Activity of *A. johnsonii*, *P. fluorescens*, and Co-culture.” Moreover, there were too many spoilage-related proteins, including thioredoxin reductase OS (6 down-regulated proteins, 5 up-regulated proteins), cysteine synthase OS (7 down-regulated proteins, 1 up-regulated proteins), and pyridoxal phosphate-dependent enzyme family protein OS. Notably, the spoilage-related proteins in *A. johnsonii*, *P. fluorescens*, and their co-culture were similar to those of *Shewanella baltica* and *P. fluorescens* ITEM 17298 (Quintieri et al., 2019).

**TABLE 4:** Quorum sensing system proteins and spoilage related proteins of *A. johnsonii*, *P. fluorescens* and co-culture.

**Function**	**Protein name**	**Accession number**	**Regulate**
QS system	AI-2E family transporter OS	A0A2S8XHJ7, A0A293PZ07, A0A2N1DUG2, A0A165YHM6, A0A2S8XHJ7,	Up
		A0A1T3AM62, A0A161GM51, A0A2K9M819, A0A2A5REX4, A0A386Y9V3, A0A1B3D5W7, A0A327NA26, A0A327MWL2, A0A423L5V9, A0A2A5R442, A0A1T2YCZ7	Down
	LuxR family transcriptional regulator OS	A0A0U3TA42	Down
Spoilage	Thioredoxin reductase OS	A0A0W8H2Q7, A0A4P6V9D1, Q4K9V2, B3PL47, A0A2K9M4Z2, A0A379J9N3	Down
		A0A379J3Q0, J2Y774, A0A2A5RE67, A0A3S4RHU3, A0A4P7I1W5	Up
	Cysteine synthase OS	A0A380UBL3, G8Q5M5, A0A379IA35, A0A506RJG5, J2MKP0, A0A3S4PFK0, A0A109KX01	Down
		A0A109LGM0	Up
	Pyridoxal-phosphate dependent enzyme family protein OS	A0A379J407, A0A3S4PDU0	Down
	Sulfate adenylyltransferase subunit 2 OS	A0A0F4TQL9	Down
	Multifunctional fusion protein OS	A0A010RIC6	Down
	Putative ABC transporter ATP-binding protein OS	A0A379JE46	
	Glycine betaine transport ATP-binding protein OpuAA OS	A0A0B7D1W3	Down
	Microcin ABC transporter ATP-binding protein OS	A0A423M0U6	Down
	ABC transporter ATP-binding protein OS	A0A379IDI7	Up
	Glutathione import ATP-binding protein GsiA OS	A0A0B7D6X0	
	Ribose import ATP-binding protein RbsA OS	A0A0P9BDW1	
	Spermidine/putrescine import ATP-binding protein PotA OS	A0A010RIC6	Down
	Spermidine/putrescine ABC transporter substrate-binding protein OS	A0A010RQY0	
	Spermidine/putrescine import ATP-binding protein PotA OS	A0A448BWD3	Up
	Transcriptional regulator, Crp/Fnr family OS	E2XZ43	Down
	Transcriptional activator CopR OS	A0A3S4PXV5	Down
	Urocanate hydratase OS	A0A0N7H007	Up

## Conclusion

This study was carried out in order to explore cultures of *A. johnsonii* and *P. fluorescens* and compare them with their co-cultured state for QS and spoilage potential by means of their proteomic profiles. The results show that the products of AHL production (C4-HSL, C6-HSL, C8-HSL), biofilm production, protease activity, and spoilage potential were at a higher level in the co-culture than those of *A. johnsonii* and *P. fluorescens* single cultures alone. The proteomic results revealed that there were differences in the proteins involved in the metabolism of amino acids, carbohydrates, energy, and translation. The differentially expressed proteins that were spoilage-related included thioredoxin reductase OS, cysteine synthase OS, and pyridoxal phosphate-dependent enzyme family protein OS, as well as specific QS system proteins of the AI-2E family transporter OS and the LuxR family transcriptional regulator OS, which could be used as biomarkers in *A. johnsonii*, *P. fluorescens*, and their co-cultured state. These results may provide an understanding of how the QS and spoilage mechanisms of *A. johnsonii*, *P. fluorescens*, and their co-cultures can be regulated in future.

## Data Availability Statement

The mass spectrometry proteomics data have been deposited to the ProteomeXchange Consortium (http://proteomecentral.proteomexchange.org/cgi/GetDataset?ID=PXD018646) and the iProX (https://www.iprox.org/page/project.html?id=IPX0002129000).

## Author Contributions

X-YW analyzed the data, wrote the manuscript, and performed the experiments. JX made suggestions for revision and guided the experiments.

## Conflict of Interest

The authors declare that the research was conducted in the absence of any commercial or financial relationships that could be construed as a potential conflict of interest.

## Supplementary Material

FIGURE S1 **(A)** KEGG pathway map of ribosome; **(B)** TCA cycle; and **(C)** oxidative phosphorylation. Proteins in the blue block belong to the experimental species. Red frames indicate up-regulated proteins, and green frames indicate down-regulated proteins.

FIGURE S2 Chromatograms of C6-HSL produced by: **(A)** *A. johnsonii*; **(B)** *P. fluorescens*; and **(C)** their co-culture. Chromatograms of C8-HSL produced by: **(D)** *A. johnsonii*; **(E)** *P. fluorescens*; and **(F)** their co-culture.

TABLE S1 KEGG pathway of the A group compared with the AP group.

TABLE S2 KEGG pathway of the P group compared with the AP group.
