# Supplementary figures and images for: Quorum Sensing System-Regulated Proteins Affect the Spoilage Potential of Co-cultured Acinetobacter johnsonii and Pseudomonas fluorescens From Spoiled Bigeye Tuna (Thunnus obesus) as Determined by Proteomic Analysis
Source: Front Microbiol. 2020 May 14;11:940. doi: 10.3389/fmicb.2020.00940 (PMC7240109; doi:10.3389/fmicb.2020.00940)

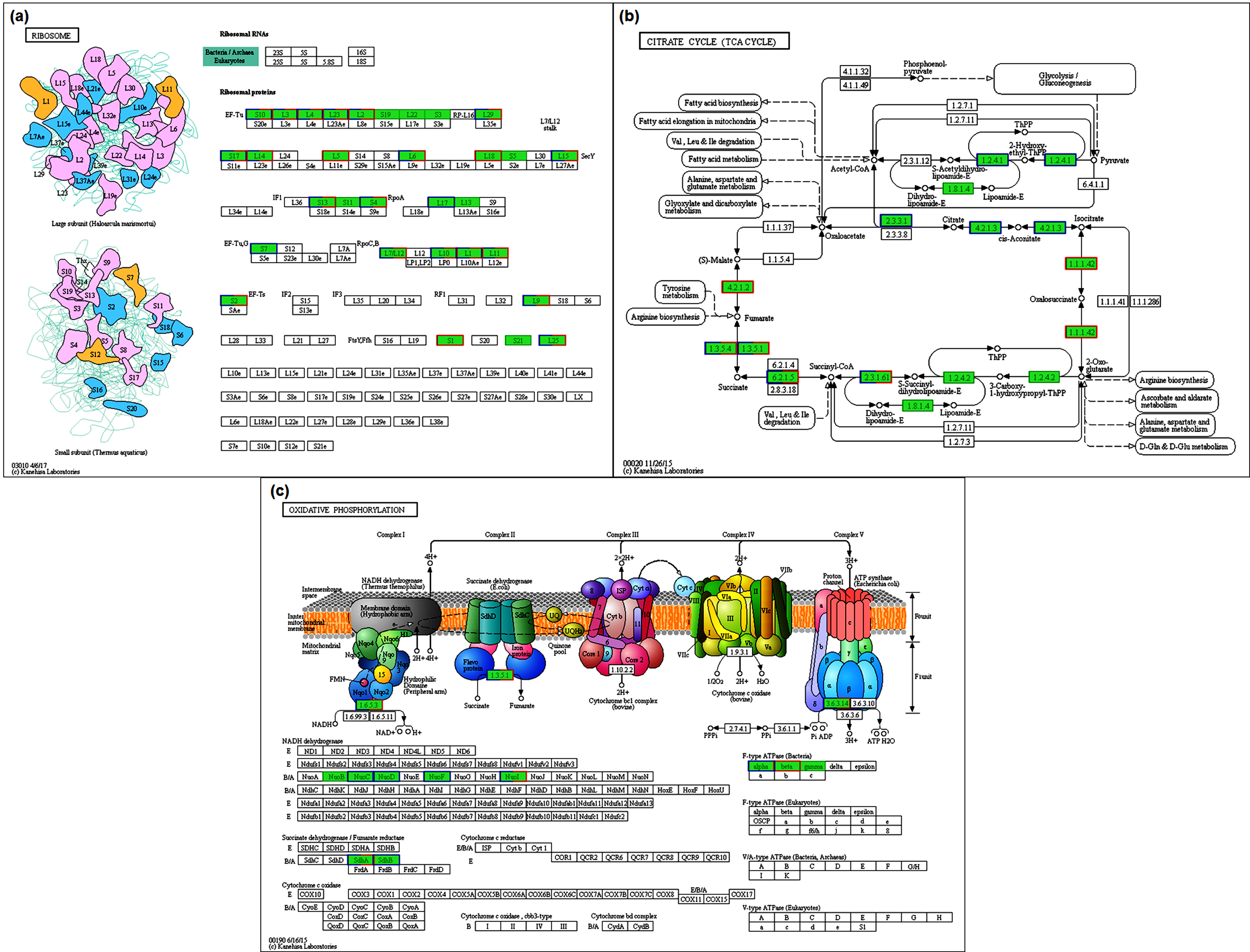

Supplement: FIGURE S1 — (A) KEGG pathway map of ribosome; (B) TCA cycle; and (C) oxidative phosphorylation. Proteins in the blue block belong to the experimental species. Red frames indicate up-regulated proteins, and green frames indicate down-regulated proteins. [file Image_1.TIF]

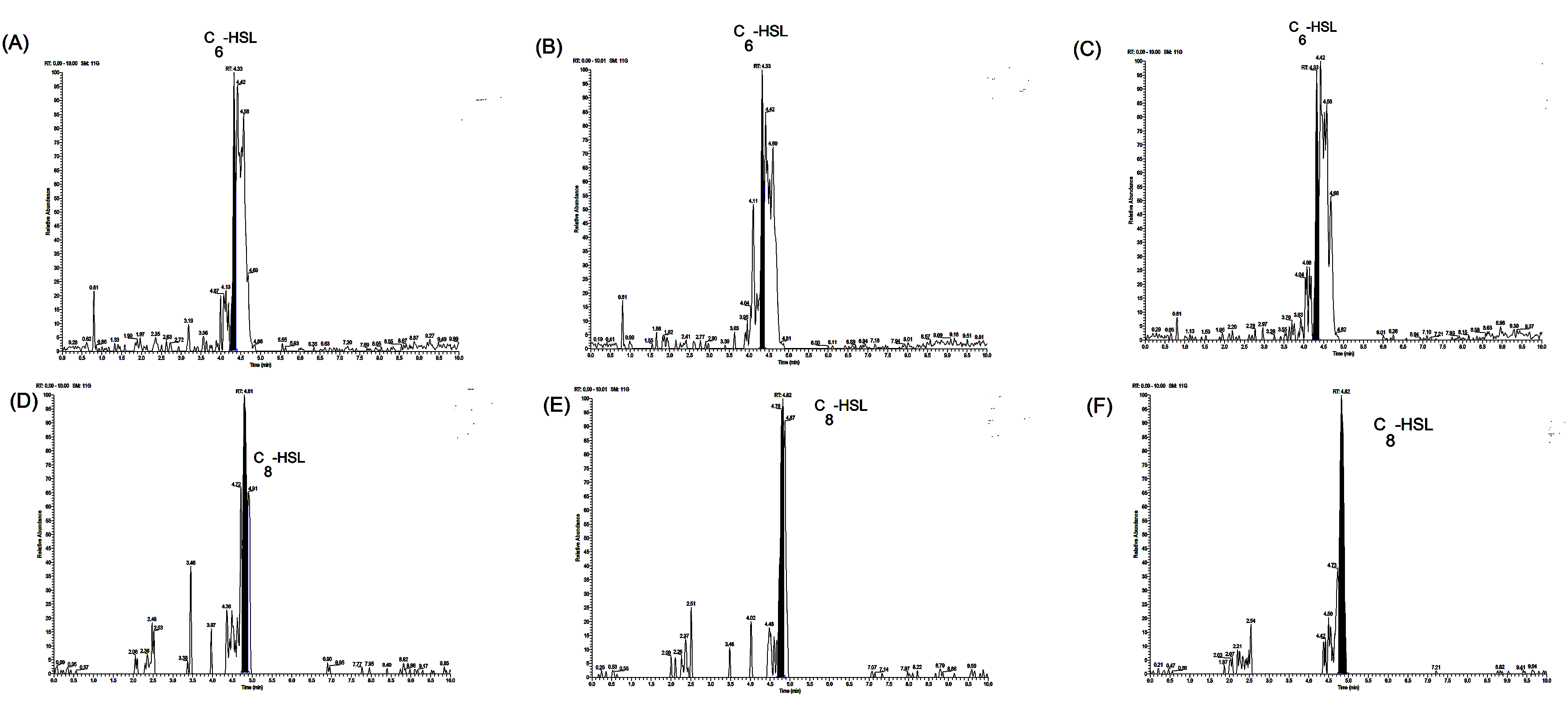

Supplement: FIGURE S2 — Chromatograms of C6-HSL produced by: (A) A. johnsonii; (B) P. fluorescens; and (C) their co-culture. Chromatograms of C8-HSL produced by: (D) A. johnsonii; (E) P. fluorescens; and (F) their co-culture. [file Image_2.TIF]
