# Supplementary material for: Quorum Sensing System-Regulated Proteins Affect the Spoilage Potential of Co-cultured Acinetobacter johnsonii and Pseudomonas fluorescens From Spoiled Bigeye Tuna (Thunnus obesus) as Determined by Proteomic Analysis
Source: Front Microbiol. 2020 May 14;11:940. doi: 10.3389/fmicb.2020.00940 (PMC7240109; doi:10.3389/fmicb.2020.00940)
Supplement: TABLE S1 — KEGG pathway of the A group compared with the AP group. [file Table_1.docx]

Table S1 KEGG pathway of A group in comparison with the AP group.

| Protein Number | Pathway ID | KEGG Description | *P* value |
| --- | --- | --- | --- |
| 21 | map03010 | Ribosome | 0.00699986 |
| 5 | map04626 | Plant-pathogen interaction | 0.014934407 |
| 5 | map00620 | Pyruvate metabolism | 0.455059186 |
| 4 | map02020 | Two-component system | 0.486957413 |
| 4 | map00270 | Cysteine and methionine metabolism | 0.998522409 |
| 3 | map04621 | NOD-like receptor signaling pathway | 0.081157998 |
| 3 | map00561 | Glycerolipid metabolism | 0.081157998 |
| 4 | map00660 | C5-Branched dibasic acid metabolism | 0.114660878 |
| 6 | map00640 | Propanoate metabolism | 0.139915542 |
| 6 | map05134 | Legionellosis | 0.139915542 |
| 10 | map00020 | Citrate cycle (TCA cycle) | 0.14493405 |
| 2 | map00220 | Arginine biosynthesis | 0.188138996 |
| 2 | map00340 | Histidine metabolism | 0.188138996 |
| 2 | map00053 | Ascorbate and aldarate metabolism | 0.188138996 |
| 2 | map02026 | Biofilm formation - Escherichia coli | 0.188138996 |
| 8 | map00720 | Carbon fixation pathways in prokaryotes | 0.217217676 |
| 3 | map05111 | Biofilm formation - Vibrio cholerae | 0.219992888 |
| 3 | map02025 | Biofilm formation - Pseudomonas aeruginosa | 0.219992888 |
| 7 | map00190 | Oxidative phosphorylation | 0.222333677 |
| 4 | map00650 | Butanoate metabolism | 0.228473008 |
| 6 | map05152 | Tuberculosis | 0.325443058 |
| 5 | map00250 | Alanine, aspartate and glutamate metabolism | 0.340826791 |
| 4 | map02024 | Quorum sensing | 0.358083422 |
| 3 | map00310 | Lysine degradation | 0.377990904 |
| 2 | map03430 | Mismatch repair | 0.402100991 |
| 2 | map03070 | Bacterial secretion system | 0.402100991 |
| 2 | map00670 | One carbon pool by folate | 0.402100991 |
| 1 | map00550 | Peptidoglycan biosynthesis | 0.43454039 |
| 1 | map00410 | beta-Alanine metabolism | 0.43454039 |
| 1 | map00590 | Arachidonic acid metabolism | 0.43454039 |
| 1 | map00903 | Limonene and pinene degradation | 0.43454039 |
| 1 | map00900 | Terpenoid backbone biosynthesis | 0.43454039 |
| 1 | map00785 | Lipoic acid metabolism | 0.43454039 |
| 1 | map00930 | Caprolactam degradation | 0.43454039 |
| 1 | map00623 | Toluene degradation | 0.43454039 |
| 1 | map05120 | Epithelial cell signaling in Helicobacter pylori infection | 0.43454039 |
| 1 | map00564 | Glycerophospholipid metabolism | 0.43454039 |
| 1 | map00040 | Pentose and glucuronate interconversions | 0.43454039 |
| 1 | map00633 | Nitrotoluene degradation | 0.43454039 |
| 1 | map00471 | D-Glutamine and D-glutamate metabolism | 0.43454039 |
| 1 | map01040 | Biosynthesis of unsaturated fatty acids | 0.43454039 |
| 1 | map00071 | Fatty acid degradation | 0.43454039 |
| 1 | map00362 | Benzoate degradation | 0.43454039 |
| 1 | map00361 | Chlorocyclohexane and chlorobenzene degradation | 0.43454039 |
| 1 | map00364 | Fluorobenzoate degradation | 0.43454039 |
| 1 | map00281 | Geraniol degradation | 0.43454039 |
| 1 | map03060 | Protein export | 0.43454039 |
| 2 | map03030 | DNA replication | 0.584209093 |
| 2 | map00261 | Monobactam biosynthesis | 0.584209093 |
| 2 | map00330 | Arginine and proline metabolism | 0.584209093 |
| 2 | map00520 | Amino sugar and nucleotide sugar metabolism | 0.584209093 |
| 7 | map03018 | RNA degradation | 0.668109242 |
| 1 | map00350 | Tyrosine metabolism | 0.680941784 |
| 1 | map01501 | beta-Lactam resistance | 0.680941784 |
| 1 | map00540 | Lipopolysaccharide biosynthesis | 0.680941784 |
| 1 | map00460 | Cyanoamino acid metabolism | 0.680941784 |
| 1 | map01503 | Cationic antimicrobial peptide (CAMP) resistance | 0.680941784 |
| 1 | map03410 | Base excision repair | 0.680941784 |
| 4 | map00630 | Glyoxylate and dicarboxylate metabolism | 0.703063729 |
| 2 | map00195 | Photosynthesis | 0.721687886 |
| 2 | map03440 | Homologous recombination | 0.721687886 |
| 1 | map04112 | Cell cycle - Caulobacter | 0.820362181 |
| 1 | map00130 | Ubiquinone and other terpenoid-quinone biosynthesis | 0.820362181 |
| 1 | map00500 | Starch and sucrose metabolism | 0.820362181 |
| 1 | map00290 | Valine, leucine and isoleucine biosynthesis | 0.820362181 |
| 1 | map00770 | Pantothenate and CoA biosynthesis | 0.820362181 |
| 1 | map00480 | Glutathione metabolism | 0.820362181 |
| 1 | map00523 | Polyketide sugar unit biosynthesis | 0.820362181 |
| 1 | map00760 | Nicotinate and nicotinamide metabolism | 0.820362181 |
| 3 | map00260 | Glycine, serine and threonine metabolism | 0.830794173 |
| 2 | map00680 | Methane metabolism | 0.884457267 |
| 1 | map00280 | Valine, leucine and isoleucine degradation | 0.899079877 |
| 8 | map00230 | Purine metabolism | 0.922074505 |
| 3 | map00010 | Glycolysis / Gluconeogenesis | 0.923964722 |
| 1 | map00300 | Lysine biosynthesis | 0.943427875 |
| 1 | map00521 | Streptomycin biosynthesis | 0.943427875 |
| 1 | map00910 | Nitrogen metabolism | 0.943427875 |
| 2 | map02010 | ABC transporters | 0.955206943 |
| 5 | map00450 | Selenocompound metabolism | 0.966065402 |
| 1 | map00380 | Tryptophan metabolism | 0.968357964 |
| 1 | map03020 | RNA polymerase | 0.968357964 |
| 2 | map00970 | Aminoacyl-tRNA biosynthesis | 0.972624731 |
| 5 | map00920 | Sulfur metabolism | 0.984647919 |
| 7 | map00240 | Pyrimidine metabolism | 0.996834303 |
