# Supplementary material for: Quorum Sensing System-Regulated Proteins Affect the Spoilage Potential of Co-cultured Acinetobacter johnsonii and Pseudomonas fluorescens From Spoiled Bigeye Tuna (Thunnus obesus) as Determined by Proteomic Analysis
Source: Front Microbiol. 2020 May 14;11:940. doi: 10.3389/fmicb.2020.00940 (PMC7240109; doi:10.3389/fmicb.2020.00940)
Supplement: TABLE S2 — KEGG pathway of the P group compared with the AP group. [file Table_2.docx]

Table S2 KEGG pathway of P group in comparison with the AP group.

| Protein Number | Pathway ID | KEGG Description | *P* value |
| --- | --- | --- | --- |
| 22 | map03010 | Ribosome | 0.000140954 |
| 3 | map00650 | Butanoate metabolism | 0.394211972 |
| 7 | map00020 | Citrate cycle (TCA cycle) | 0.45072927 |
| 7 | map00240 | Pyrimidine metabolism | 0.977091907 |
| 5 | map00920 | Sulfur metabolism | 0.940903642 |
| 5 | map00270 | Cysteine and methionine metabolism | 0.968026134 |
| 2 | map00260 | Glycine, serine and threonine metabolism | 0.905101241 |
| 2 | map00030 | Pentose phosphate pathway | 0.473509865 |
| 2 | map00630 | Glyoxylate and dicarboxylate metabolism | 0.935377634 |
| 1 | map03420 | Nucleotide excision repair | 0.962163604 |
| 4 | map03018 | RNA degradation | 0.929509411 |
| 2 | map00220 | Arginine biosynthesis | 0.136599181 |
| 2 | map00460 | Cyanoamino acid metabolism | 0.136599181 |
| 3 | map00330 | Arginine and proline metabolism | 0.145586705 |
| 5 | map02010 | ABC transporters | 0.205362422 |
| 7 | map00720 | Carbon fixation pathways in prokaryotes | 0.226598026 |
| 4 | map00680 | Methane metabolism | 0.232973493 |
| 3 | map00310 | Lysine degradation | 0.26659497 |
| 3 | map00521 | Streptomycin biosynthesis | 0.26659497 |
| 3 | map00660 | C5-Branched dibasic acid metabolism | 0.26659497 |
| 2 | map00670 | One carbon pool by folate | 0.309548285 |
| 2 | map00480 | Glutathione metabolism | 0.309548285 |
| 2 | map00523 | Polyketide sugar unit biosynthesis | 0.309548285 |
| 2 | map00500 | Starch and sucrose metabolism | 0.309548285 |
| 1 | map00564 | Glycerophospholipid metabolism | 0.370473538 |
| 1 | map00750 | Vitamin B6 metabolism | 0.370473538 |
| 1 | map00900 | Terpenoid backbone biosynthesis | 0.370473538 |
| 1 | map00633 | Nitrotoluene degradation | 0.370473538 |
| 1 | map00590 | Arachidonic acid metabolism | 0.370473538 |
| 1 | map00903 | Limonene and pinene degradation | 0.370473538 |
| 1 | map00410 | beta-Alanine metabolism | 0.370473538 |
| 1 | map00051 | Fructose and mannose metabolism | 0.370473538 |
| 1 | map01040 | Biosynthesis of unsaturated fatty acids | 0.370473538 |
| 1 | map04122 | Sulfur relay system | 0.370473538 |
| 1 | map00071 | Fatty acid degradation | 0.370473538 |
| 1 | map00785 | Lipoic acid metabolism | 0.370473538 |
| 1 | map00052 | Galactose metabolism | 0.370473538 |
| 1 | map00362 | Benzoate degradation | 0.370473538 |
| 1 | map00930 | Caprolactam degradation | 0.370473538 |
| 1 | map00281 | Geraniol degradation | 0.370473538 |
| 1 | map00730 | Thiamine metabolism | 0.370473538 |
| 1 | map05120 | Epithelial cell signaling in Helicobacter pylori infection | 0.370473538 |
| 4 | map00250 | Alanine, aspartate and glutamate metabolism | 0.442639501 |
| 4 | map00640 | Propanoate metabolism | 0.442639501 |
| 4 | map00970 | Aminoacyl-tRNA biosynthesis | 0.542521853 |
| 1 | map03410 | Base excision repair | 0.604347894 |
| 1 | map00350 | Tyrosine metabolism | 0.604347894 |
| 1 | map02026 | Biofilm formation - Escherichia coli | 0.604347894 |
| 1 | map00340 | Histidine metabolism | 0.604347894 |
| 1 | map00525 | Acarbose and validamycin biosynthesis | 0.604347894 |
| 2 | map00300 | Lysine biosynthesis | 0.611453129 |
| 9 | map00230 | Purine metabolism | 0.622294925 |
| 3 | map05134 | Legionellosis | 0.711553525 |
| 2 | map00380 | Tryptophan metabolism | 0.720073707 |
| 1 | map04112 | Cell cycle - Caulobacter | 0.751747698 |
| 1 | map03430 | Mismatch repair | 0.751747698 |
| 1 | map00400 | Phenylalanine, tyrosine and tryptophan biosynthesis | 0.751747698 |
| 1 | map00760 | Nicotinate and nicotinamide metabolism | 0.751747698 |
| 1 | map04621 | NOD-like receptor signaling pathway | 0.751747698 |
| 6 | map00450 | Selenocompound metabolism | 0.770653171 |
| 3 | map00620 | Pyruvate metabolism | 0.78399567 |
| 2 | map02024 | Quorum sensing | 0.802046857 |
| 3 | map05152 | Tuberculosis | 0.840818126 |
| 3 | map00010 | Glycolysis / Gluconeogenesis | 0.840818126 |
| 1 | map02025 | Biofilm formation - Pseudomonas aeruginosa | 0.844493642 |
| 1 | map00710 | Carbon fixation in photosynthetic organisms | 0.844493642 |
| 1 | map00520 | Amino sugar and nucleotide sugar metabolism | 0.844493642 |
| 1 | map00261 | Monobactam biosynthesis | 0.844493642 |
| 1 | map00280 | Valine, leucine and isoleucine degradation | 0.844493642 |
| 1 | map03030 | DNA replication | 0.844493642 |
| 1 | map05111 | Biofilm formation - Vibrio cholerae | 0.844493642 |
| 2 | map02020 | Two-component system | 0.862090638 |
| 3 | map00190 | Oxidative phosphorylation | 0.884323953 |
| 1 | map03440 | Homologous recombination | 0.902753771 |
| 1 | map00195 | Photosynthesis | 0.902753771 |
| 1 | map04626 | Plant-pathogen interaction | 0.902753771 |
| 1 | map00910 | Nitrogen metabolism | 0.902753771 |
